# Supplementary material for: Fused in sarcoma (FUS) inhibits milk production efficiency in mammals
Source: Nat Commun. 2024 May 10;15:3953. doi: 10.1038/s41467-024-48428-5 (PMC11087553; doi:10.1038/s41467-024-48428-5)
Supplement: Supplementary file 1 — Supplementary information [file 41467_2024_48428_MOESM1_ESM.pdf]

Supplementary Fig. 1

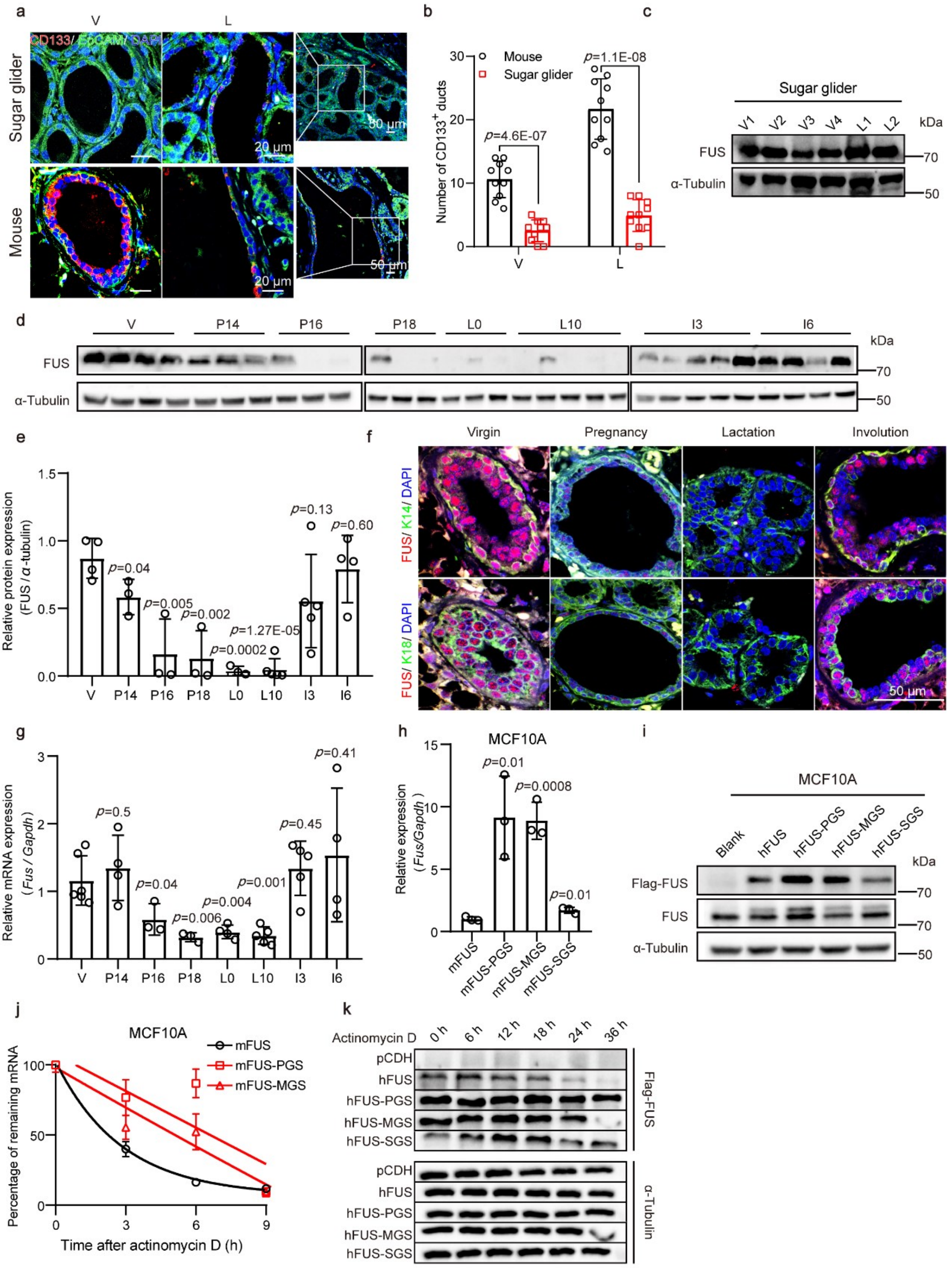

Supplementary Fig. 1 FUS expression during mammary gland development.

(a) Immunofluorescence staining of mammary glands of sugar glider and mice for CD133 (red), EpCAM (green), and DAPI (blue) at virgin and lactation. The experiments were performed to have three biological replicates independently with

similar results. Scale bar: 50 μm; 20 μm. (b) Statistical analysis of number of $CD133^+$ mammary gland ducts in sugar gliders and mice. (c) The protein expression of FUS in sugar gliders at virgin and lactation stages. (d-e) Western blot analysis (d) and relative quantification (e) of FUS protein levels at different developmental stages (3-5 replicates per stage) in mice. (f) Immunofluorescence staining of mammary glands of mice for FUS (red), K14 (green, upper, cytokeratin 14-labeled myoepithelial cell layers), K18 (green, bottom, cytokeratin 18-labeled luminal cell layers), and DAPI (blue) at different developmental stages. The experiments were performed to have three biological replicates independently with similar results. Scale bar: 50 μm. (g) Relative mRNA expression levels of *Fus* at different developmental stages (3-6 replicates per stage) in mice. (h-i) RT-qPCR analysis of *Fus* mRNA expression (h) and western blot analysis of FUS protein levels (i) in MCF10A cells treated after overexpression of indicated vectors. (j) qRT-PCR analysis of *Fus* mRNA expression in MCF10A cells treated with actinomycin D after overexpression of FUS full-length (hFUS), and FUS mutant (hFUS-PGS and hFUS-MGS). (k) Western blot analysis of Flag-FUS protein levels in HC11 cells treated with actinomycin D after overexpression of pCDH-3×Flag (pCDH), FUS full-length (hFUS), and FUS mutant (hFUS-PGS, hFUS-MGS, and hFUS-SGS). The statistical test used was two-sided in graphs b, e, g and h. *P*-values were indicated in the chart (b,e, g, h). Source data are provided as a Source Data file.

Supplementary Fig. 2

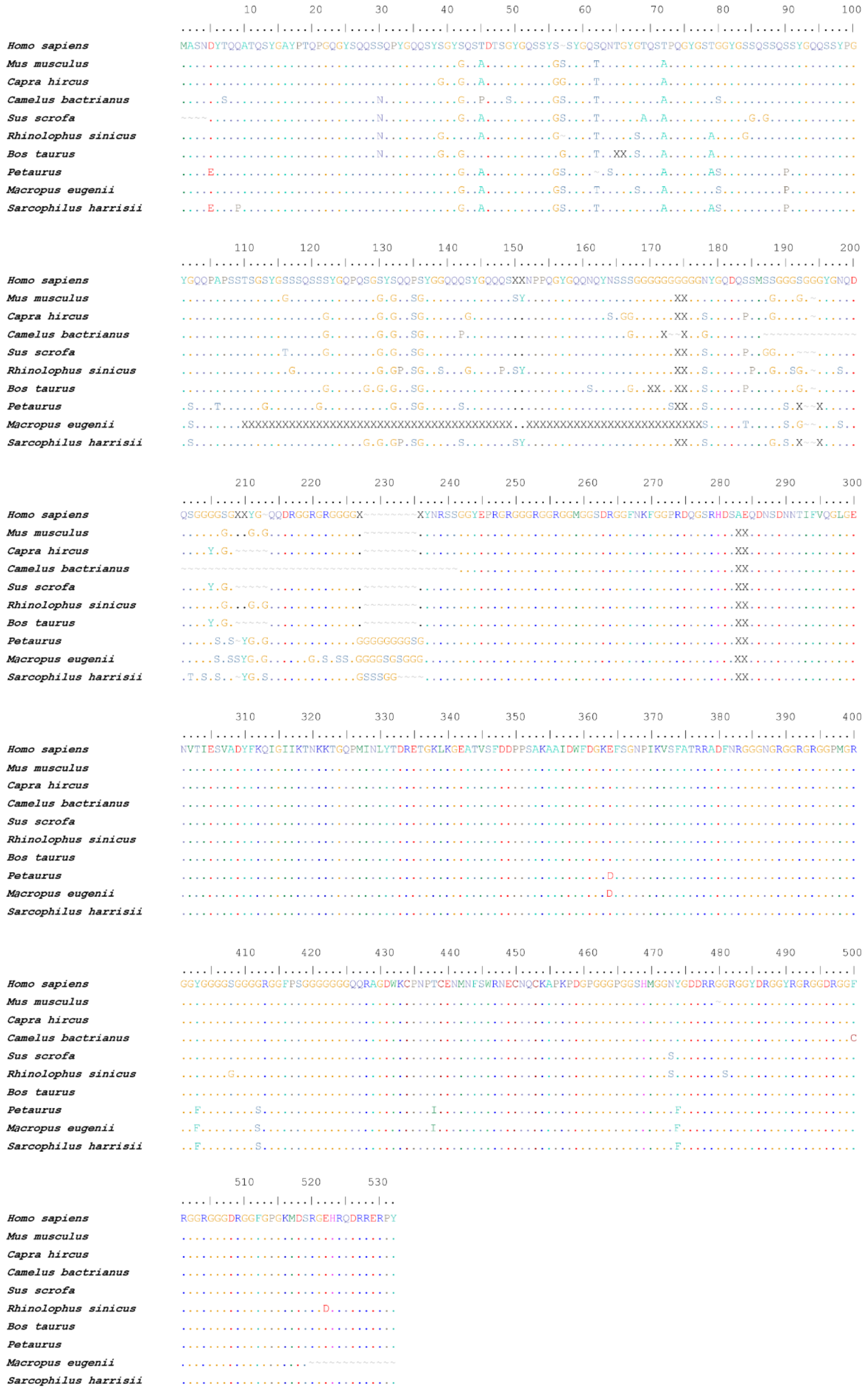

Supplementary Fig. 2 Multiple alignments of FUS amino acid sequences between eutherians and marsupials. Multiple alignments, containing a total of 532 amino acids.

Supplementary Fig. 3

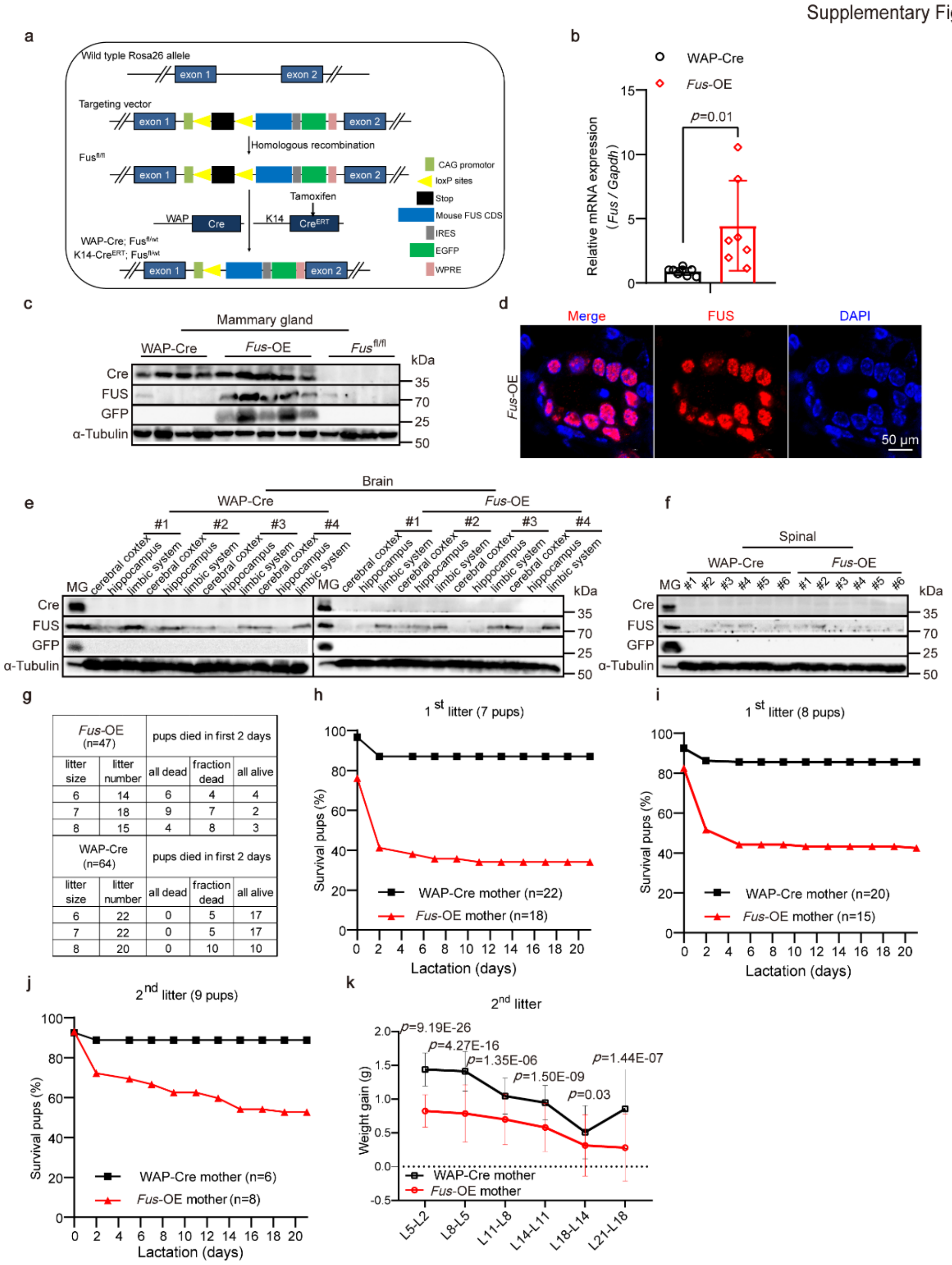

Fus-OE (n=47)		pups died in first 2 days		
litter size	litter number	all dead	fraction dead	all alive
6	14	6	4	4
7	18	9	7	2
8	15	4	8	3
WAP-Cre (n=64)		pups died in first 2 days		
litter size	litter number	all dead	fraction dead	all alive
6	22	0	5	17
7	22	0	5	17
8	20	0	10	10

Supplementary Fig. 3 FUS overexpression induces failure of both pup viability and growth.

(a) Mating strategy of *Fus*-OE mice. Mice homozygous for a floxed allele of *Fus* (*Fus*$^{fl/fl}$ mice) were crossed with WAP-Cre or K14-Cre^{ERT} transgenic mice. Pregnant (*Fus*$^{fl/fl}$/K14-Cre^{ERT}) mice were injected intraperitoneally with 240 mg/kg tamoxifen/mouse body weight, divided into three doses once every other day.

Tamoxifen was dissolved in sunflower oil containing 10% ethanol at a final concentration of 20 mg/ml [1, 2]. (b) *Fus* mRNA levels in mammary glands of WAP-Cre and *Fus*-OE mice at L0. n = 7 mice. (c, e-f) Western blot analysis of Cre, FUS, GFP expression levels in mammary gland (c), brain (including cerebral cortex and limbic system) (e) and spinal (f) in WAP-Cre, *Fus*-OE and *Fus*$^{fl/fl}$ mice. MG: mammary gland tissue of *Fus*-OE mice. (d) Immunofluorescence staining of mammary glands of *Fus*-OE mice for FUS (red) and DAPI (blue) at lactation. The experiments were performed to have three biological replicates independently with similar results. Scale bar: 50 μm. (g) Litter pup deaths (all dead or a fraction of each litter) from *Fus*-OE and WAP-Cre female mice during first 2 d. (h-j) Pup survival rates for *Fus*-OE and WAP-Cre mice during first lactation, with seven (h) or eight pups (i) per litter, and during second lactation, with nine pups (j) per litter. (k) Weight gain of pups after litter size adjustment to seven on L2 during second lactation. The statistical test used was two-sided in graphs b and k. *P*-values were indicated in the chart (b, k). Source data are provided as a Source Data file.

Supplementary Fig. 4

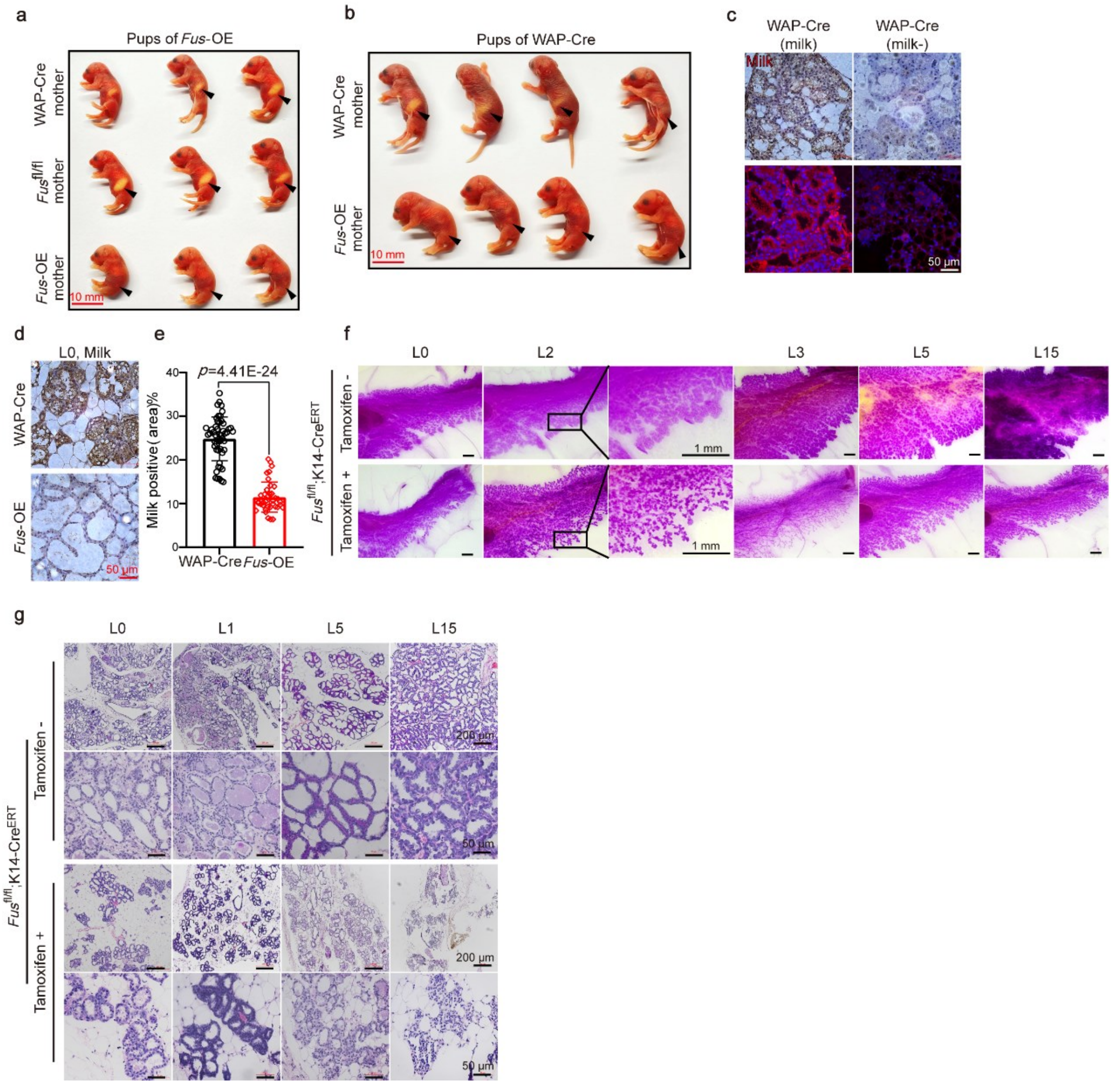

Supplementary Fig. 4 *Fus* overexpression leads to low milk production.

(a-b) Gross appearance of neonates born to *Fus*-OE (S4A) and WAP-Cre mothers (S4B) but fed by WAP-Cre, *Fus*$^{fl/fl}$, or *Fus*-OE mothers. Representative stomach (arrowheads) of neonate is shown. Scale bar: 10 mm. (c) Immunohistochemical (upper) and immunofluorescence staining (lower) of mammary gland sections of female WAP-Cre mice to visualize milk (red) and DAPI-stained cell nuclei (blue) in samples collected from mice that either produced (left) or did not produce milk (right). The experiments were performed to have three biological replicates independently with similar results. Scale bar: 50 μm. (d-e) Immunohistochemical analysis (d) and statistics (e) of milk in

Fus-OE and WAP-Cre mice at L0. Scale bar: 50 μm. The statistical test used was two-sided. *P*-values were indicated in the chart. (f-g) Whole-mount carmine staining (f) and representative H&E staining (g) of mammary glands of K14-Cre^{ERT}; *Fus*$^{fl/fl}$ mice induced by tamoxifen. Magnified areas are shown in black boxes. The experiments were performed to have three biological replicates independently with similar results. Scale bar: 1 mm (f); 200 μm (upper); 50 μm (bottom) (g). Data are means ± SD. Unpaired *t*-test was used to evaluate statistical significance. Source data are provided as a Source Data file.

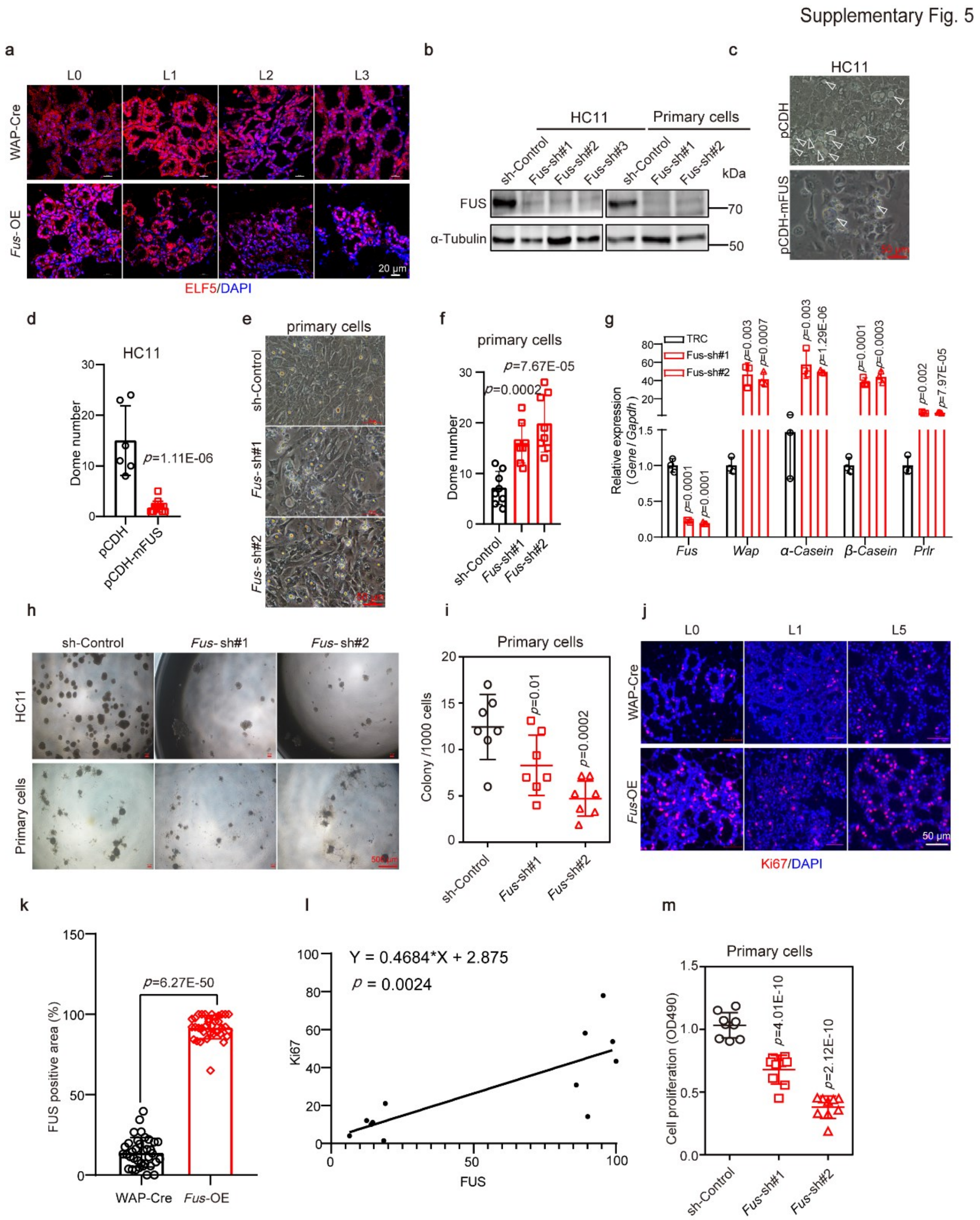

Supplementary Fig. 5 FUS regulates coordination between cell cycle exit of MEC and binds to *p57Kip2* mRNA.

(a) Immunofluorescence staining of mammary glands of ELF5 (red) and DAPI (bule) at L0, L1, L2, and L3 from WAP-Cre and *Fus*-OE mice (three in each group). The experiments were performed to have three biological replicates independently with

similar results. Scale bar: 50 μm. (b) Western blot analysis of *Fus* knockout efficiency in HC11 and primary cells after treatment with indicated shRNA vectors. (c-d) Representative morphological images (c) and statistics (d) of dome formation during *in vitro* differentiation of HC11 cells expressed with indicated vectors. Arrowhead indicates dome structure. (e-f) Representative morphological images (e) and statistics (f) of dome formation in mouse primary cell culture upon *Fus* knockdown. (g) Relative mRNA expression of milk protein-related genes in HC11 cells after *Fus* knockdown, as determined by RT-qPCR. (h) Representative clone assay images of HC11 and primary cells expressed with indicated vectors. Scale bar: 500 μm. (i) Colony number per 1 000 primary cells. (j) Immunofluorescence staining of Ki67 (red) and DAPI (blue) in mammary glands from WAP-Cre and *Fus*-OE mice at L0, L1, and L5. Representative images are from five mice for each sample. The experiments were performed to have three biological replicates independently with similar results. Scale bar: 50 μm. (k-l) Statistics of FUS (k) and correlation analysis of FUS and Ki67 (l) at L0. (m) Analysis of primary cell proliferation upon *Fus* knockdown by MTS assay. Data are means ± SD. The statistical test used was two-sided in graphs d, f, g, i, k and m. *P*-values were indicated in the chart (d, f, g, i, k, m). Source data are provided as a Source Data file.

Supplementary Fig. 6

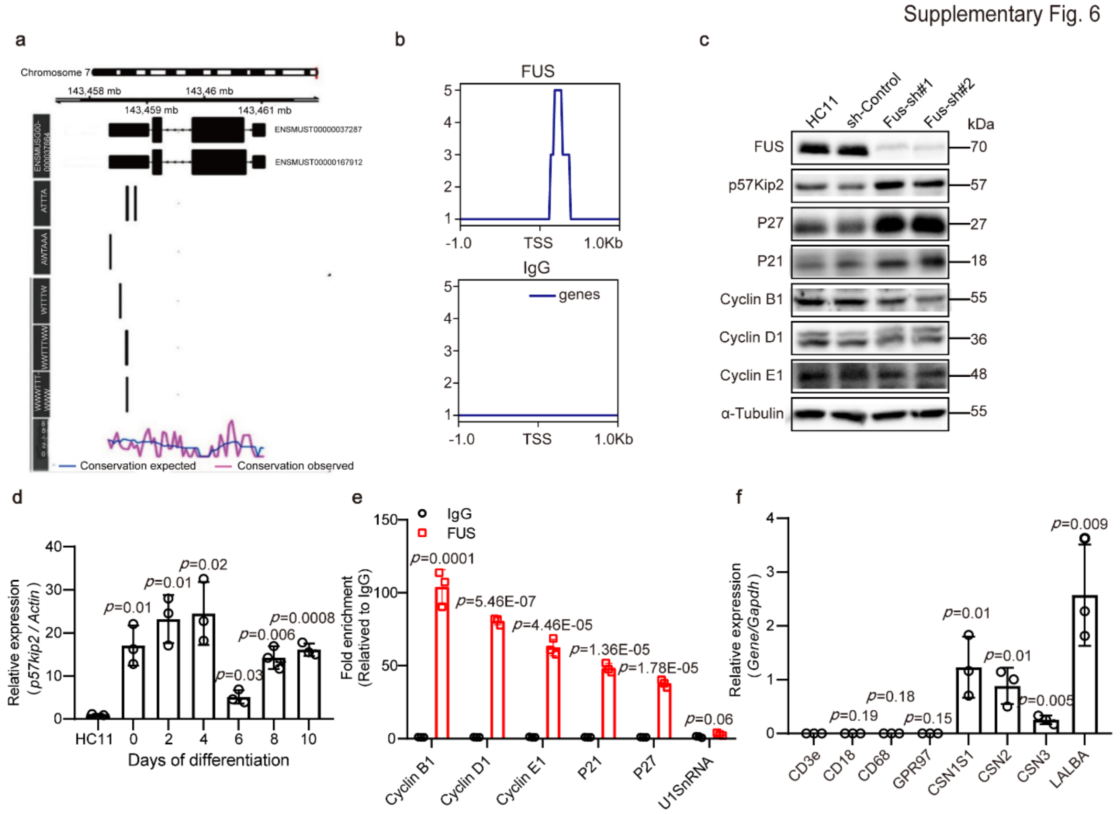

Supplementary Fig. 6 FUS directly binds to *p57Kip2* mRNA and decreases its stability.

(a) Prediction results of FUS binding to *p57Kip2* mRNA (http://nibiru.tbi.univie.ac.at). (b) RIP-Seq assay for analysis of interaction between FUS protein and *p57Kip2* mRNA in HC11 cells. (c) Western blot analysis of indicated protein levels in HC11 cells upon *Fus* knockdown. (d) Relative mRNA expression levels of *p57Kip2* during *in vitro* differentiation of HC11 cells at indicated time points. (e) RIP-qPCR analysis of interaction between FUS protein and indicated mRNA in HC11 cells. U1SnRNA was applied as a negative control. (f) Relative mRNA expression levels of markers in MFGs (n = 3). Specific markers for lymphocytes (CD3e), macrophages (CD18 and CD68), polymorphonuclear neutrophils (CD18 and GPR97), and milk protein-encoding genes (CSN1S1, CSN2, CSN3, and LALBA) are shown. Data are means ± SD of three

independent experiments. The statistical test used was two-sided in graphs d-f. *P*-values were indicated in the chart (d-f). Source data are provided as a Source Data file.

Supplementary Fig. 7

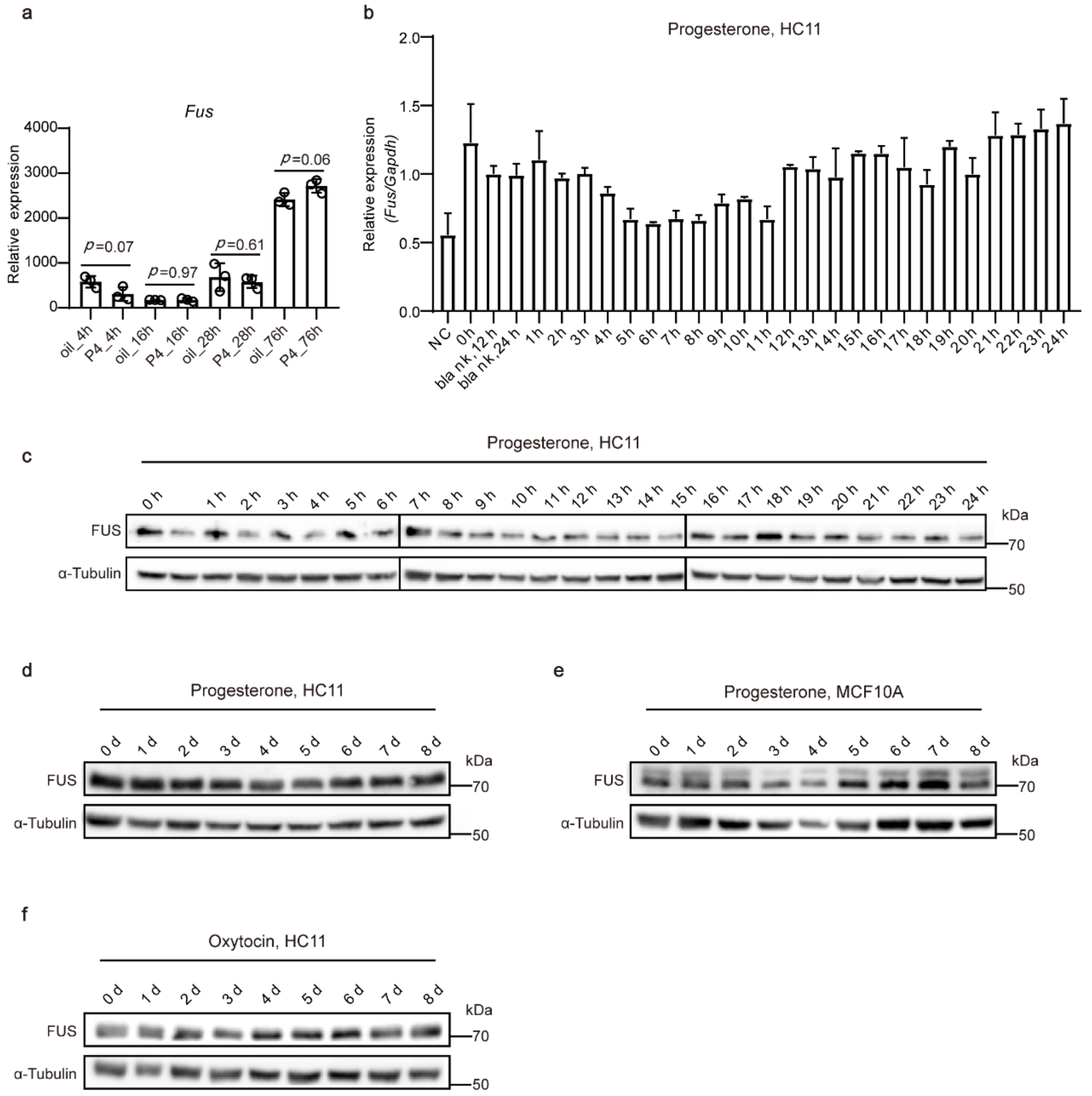

Supplementary Fig. 7 FUS expression is not regulated by progesterone or oxytocin. (a) Related FUS expression after exogenous progesterone injection in ovariectomized mice (RNA-seq data from [3]). The statistical test used was two-sided in graphs. *P*-values were indicated in the chart. (b-e) RT-qPCR analysis of *Fus* mRNA expression (b) and western blot analysis of FUS protein levels (c-e) after HC11 (c-d) and MCF10A cells (e) were treated with progesterone for indicated times. (f) Western blot analysis of FUS protein levels after HC11 cells were treated with oxytocin for 0-8 d. Source data are provided as a Source Data file.

Supplementary Table 1: 26 DEGs during lactation in comparison with those during virginity in cattle, pigs and mice.

Gene.symbol	Gene.ID	logFC	P.Value	adj.P.Val
Actc1	11464	-3.31424	0.00014	0.079159
Aspm	12316	3.932625	0.000188	0.083726
Dlg3	53310	2.971525	0.000245	0.094613
Crabp2	12904	3.775568	0.00034	0.097734
Nos3	18127	-2.98187	0.000361	0.097734
Tfap2c	21420	2.715982	0.000459	0.097734
Bub1	12235	2.07832	0.000545	0.097734
Galnt3	14425	1.85863	0.000856	0.100856
Ckap2	80986	1.85095	0.001749	0.123856
Rhou	69581	2.059171	0.001971	0.12653
Pvalb	19293	-5.88644	0.002545	0.133406
Kif11	16551	1.959696	0.002891	0.136007
Kif22	110033	1.523477	0.004164	0.144362
Timp2	21858	1.571891	0.005726	0.158157
Cdc20	107995	1.833491	0.006414	0.162464
Cytip	227929	0.933083	0.009561	0.17347
Cdca7	66953	1.851696	0.009839	0.174583
Hells	15201	1.566578	0.010691	0.178573
Cp	12870	0.854981	0.014678	0.195417
Kif20a	19348	3.549763	0.016187	0.201218
Racgap1	26934	1.330023	0.018611	0.208268
Ccnf	12449	0.755559	0.019369	0.209404
Ahr	11622	1.602942	0.020374	0.214524
Kif23	71819	0.871089	0.023161	0.222732
Fus	233908	-1.30504	0.02638	0.383773
Nfil3	18030	-0.86644	0.034831	0.2615

Supplementary Table 2. Primers and shRNA sequences used in this paper.

Primers	Sequence
sh-Mus-Fus-1F	CCGGCCCAGTGTTACCCTTGTTATTCTCGAGAATAACAAG GGTAACACTGGGTTTTTG
sh-Mus-Fus-1R	AATTCAAAAACTTTGTTCGATTTACAGAATACTCGAGTAT TCTGTAAATCGAACAAAG
sh-Mus-Fus-2F	CCGGCCAACAGAGTTACAGTGGTTACTCGAGTAACCACT GTAACTCTGTTGGTTTTTG
sh-Mus-Fus-2R	AATTCAAAAACCAACAGAGTTACAGTGGTTACTCGA GTAACCACTGTAACTCTGTTGG
sh-Mus-Fus-3F	CCGGCCTAGGCGAGAATGTTACAATCTCGAGATTGTAAC ATTCTCGCCTAGGTTTTTG
sh-Mus-Fus-3R	AATTCAAAAACCTAGGCGAGAATGTTACAATCTCGA GATTGTAACATTCTCGCCTAGG
Mus-β-actin-F	GGCTGTATTCCCCTCCATCG
Mus-β-actin-R	CCAGTTGGTAACAATGCCATGT
Mus-Gapdh-F	AGGTCGGTGTGAACGGATTTG
Mus-Gapdh-R	TGTAGACCATGTAGTTGAGGTCA
Mus-Wap-F	TATCATCTGCCAAACCAACG
Mus-Wap-R	GGTCGCTGGAGCATTCTATC
Mus-β-Casein-F	GGTGAATCTCATGGGACAGC
Mus-β-Casein-R	CACAGGGGGTTGAGCAATAG
Mus-Prlr-F	CACTTGCTTACATGCTGCTTG
Mus-Prlr-R	CAGGTGGTGACTGTCCATTCA
Mus-Fus-F	TGGCAAGTTGAAGGGTGA
Mus-Fus-R	GGCGGGTAGCAAATGAAA
Mus-p57Kip2-F	CCAGCCTCTCTCGGGGATTC
Mus-p57Kip2-F	CCGTTAGCCTCTAAACTAACTCA
Homo-Fus-F	CAATAAATTTGGTGGCCCTCGG
Homo-Fus-R	ATCATGGGCTGTCCCGTTTT
Homo-Gapdh-F	CAAATTCCATGGCACCGTCA
Homo-Gapdh-R	GACTCCACGACGTACTCAGC
Petaurus-Gapdh-F	GTGGAGTAGACGTTGTGGCCAT

Petaurus-Gapdh-R	GGCATGGACTGTAGTCATGAGTCC
Petaurus-Fus-F	CTCAGTCAGCTCCCCAAGGATAT
Petaurus-Fus-R	CCCCACTGCTGCTGTTATACTGG
Mus-Stat5-F	GCCGTGGGATGCTATTGA
Mus-Stat5-R	GGTGCTCCGCCTTCTTCT
Mus-Elf5-F	TTCGCTCGCAAGGTTACTC
Mus-Elf5-R	TATCTCAGGGCTCGGCTC
Mus-Nfib-F	CTTTGTGCTTACCGTGAC
Mus-Nfib-R	AGTTGACTCCAGTTCCCT
Homo-CD3e-F	CCTCTTATCAGTTGGCGTTTGG
Homo-CD3e-R	TTCAGTGACAGGTGATCCTCA
Homo-CD18-F	TGCGTCCTCTCTCAGGAGTG
Homo-CD18-R	GGTCCATGATGTCGTCAGCC
Homo-CD68-F	GGAAATGCCACGGTTCATCCA
Homo-CD68-R	TGGGGTTCAGTACAGAGATGC
Homo-Gpr97-F	CGAAGGGCCAAGAAACACCT
Homo-Gpr97-R	CGTAGTTTAGCCAGTATCTCTGC
Homo-CSN1S1-F	CATGCCCAGGAGCAAATTCG
Homo-CSN1S1-R	TTGGAGATGTCGGAAAACGGT
Homo-CSN2-F	CCCTGTGGTCTGTTCCTCAG
Homo-CSN2-R	TGGGTGGGGTTAAGTAGAAGTTC
Homo-CSN3-F	CCAATTTGTACCAACGTAGACCA
Homo-CSN3-R	GGGCATGTGGCCTAACTACAG
Homo-LALBA-F	GGCATCGCTTTGCCTGAATTG
Homo-LALBA-R	TTGGCACACATTATGTCATCAGT
U1snRNA-F	GGGAGATACCATGATCACGAAGGT
U1snRNA-R	CCACAAATTATGCAGTCGAGTTTCCC

References

1. McLellan, M.A., N.A. Rosenthal, and A.R. Pinto. Cre-loxP-Mediated Recombination: General Principles and Experimental Considerations. *Current Protocols In Mouse Biology* **7** (2017).
2. Kos, C.H. Cre/loxP system for generating tissue-specific knockout mouse models. *Nutrition Reviews* **62**, 243-246 (2004).
3. Fernandez-Valdivia, R., et al. Transcriptional response of the murine mammary gland to acute progesterone exposure. *Endocrinology* **149**, 6236-6250 (2008).
