# Supplementary material for: Fused in sarcoma (FUS) inhibits milk production efficiency in mammals
Source: Nat Commun. 2024 May 10;15:3953. doi: 10.1038/s41467-024-48428-5 (PMC11087553; doi:10.1038/s41467-024-48428-5)
Supplement: Supplementary file 3 — Description of Additional Supplementary Files [file 41467_2024_48428_MOESM3_ESM.docx]

**Description of Additional Supplementary Files:**

**Supplementary Data 1:**

2214 genes exhibit significant expression difference between virgin and lactation in mouse mammary gland but no significant changes in sugar glider mammary gland, related to Figure 2.

**Supplementary Data 2:**

5 genes exhibit significant expression difference between virgin and lactation in 3 Eutheria species mammary glands but no significant changes in sugar glider mammary gland, related to Figure 2.

**Supplementary Data 3:**

mRNAs associated with cell cycle of FUS binding by online prediction, related to Figure 6.
