# Supplementary material for: Fused in sarcoma (FUS) inhibits milk production efficiency in mammals
Source: Nat Commun. 2024 May 10;15:3953. doi: 10.1038/s41467-024-48428-5 (PMC11087553; doi:10.1038/s41467-024-48428-5)
Supplement: Supplementary file 7 — Reporting Summary [file 41467_2024_48428_MOESM7_ESM.pdf]

nature portfolio

Corresponding author(s): Baowei Jiao

Last updated by author(s): Apr 22, 2024

Reporting Summary

Nature Portfolio wishes to improve the reproducibility of the work that we publish. This form provides structure for consistency and transparency in reporting. For further information on Nature Portfolio policies, see our Editorial Policies and the Editorial Policy Checklist.

Statistics

For all statistical analyses, confirm that the following items are present in the figure legend, table legend, main text, or Methods section.

n/a	Confirmed	
☐	☒	The exact sample size (*n*) for each experimental group/condition, given as a discrete number and unit of measurement
☐	☒	A statement on whether measurements were taken from distinct samples or whether the same sample was measured repeatedly
☐	☒	The statistical test(s) used AND whether they are one- or two-sided<br>*Only common tests should be described solely by name; describe more complex techniques in the Methods section.*
☒	☐	A description of all covariates tested
☒	☐	A description of any assumptions or corrections, such as tests of normality and adjustment for multiple comparisons
☐	☒	A full description of the statistical parameters including central tendency (e.g. means) or other basic estimates (e.g. regression coefficient) AND variation (e.g. standard deviation) or associated estimates of uncertainty (e.g. confidence intervals)
☐	☒	For null hypothesis testing, the test statistic (e.g. *F*, *t*, *r*) with confidence intervals, effect sizes, degrees of freedom and *P* value noted<br>*Give P values as exact values whenever suitable.*
☒	☐	For Bayesian analysis, information on the choice of priors and Markov chain Monte Carlo settings
☒	☐	For hierarchical and complex designs, identification of the appropriate level for tests and full reporting of outcomes
☒	☐	Estimates of effect sizes (e.g. Cohen's *d*, Pearson's *r*), indicating how they were calculated

Our web collection on statistics for biologists contains articles on many of the points above.

Software and code

Policy information about availability of computer code

Data collection	Microscopy images were taken using NIS-Elements F 4.0. qRT-PCR data were collected using QuantStudio 3. Western blot images were taken using SageCapture software. Electron micrographs were captured by JEM 1400 Plus.
Data analysis	Graphs and statistical analyses were performed using GraphPad Prism 8. STAR software STAR_2.4.2a (Dobin A et al., 2013), FeatureCounts v1.4.6-p5 (Liao Y et al., 2014) and edgeR package 1.6 (Robinson MD et al., 2014) were used for RNAseq analysis which is briefly described in the manuscript (Methods). Microscopy images were analyzed using Image-Pro Plus 5.

For manuscripts utilizing custom algorithms or software that are central to the research but not yet described in published literature, software must be made available to editors and reviewers. We strongly encourage code deposition in a community repository (e.g. GitHub). See the Nature Portfolio guidelines for submitting code & software for further information.

Data

Policy information about availability of data

All manuscripts must include a data availability statement. This statement should provide the following information, where applicable:

- Accession codes, unique identifiers, or web links for publicly available datasets
- A description of any restrictions on data availability
- For clinical datasets or third party data, please ensure that the statement adheres to our policy

All data used in this study are available within the Article and Supplementary information, or available from the corresponding authors on reasonable request.

Source data are provided as Source Data file. Source data are provided with this paper. The raw transcriptomics data produced and analyzed in this study have been deposited in the Genome Sequence Archive in National Genomics Data Center, China National Center for Bioinformation / Beijing Institute of Genomics, Chinese Academy of Sciences that are publicly accessible at https://bigd.big.ac.cn/gsa/browse/CRA014528. The accession code is GSA: CRA014528.

Research involving human participants, their data, or biological material

Policy information about studies with human participants or human data. See also policy information about sex, gender (identity/presentation), and sexual orientation and race, ethnicity and racism.

Reporting on sex and gender	In this study, fresh human milk was obtained from 55 healthy female who gave birth to full-term babies 3-5 days after delivery.
Reporting on race, ethnicity, or other socially relevant groupings	Race, ethnicity, or other socially relevant groups were not involved in this study.
Population characteristics	Population are 20-40-old healthy female who gave birth to term infants, which have similar time span of mother pregnancy, times of gestation and pregnancy.
Recruitment	Participants were recruited voluntarily without any possible self-selection bias or other biases. Fresh human milk were obtained from 55 healthy female on days 3-5 postpartum who gave birth to term infants.
Ethics oversight	This project was approved by the Ethics Committee of Weifang People's Hospital or Luoyang Maternal and Child Health Hospital. Written informed consent was obtained from all participants.

Note that full information on the approval of the study protocol must also be provided in the manuscript.

Field-specific reporting

Please select the one below that is the best fit for your research. If you are not sure, read the appropriate sections before making your selection.

☒ Life sciences ☐ Behavioural & social sciences ☐ Ecological, evolutionary & environmental sciences

For a reference copy of the document with all sections, see nature.com/documents/nr-reporting-summary-flat.pdf

Life sciences study design

All studies must disclose on these points even when the disclosure is negative.

Sample size	The sample size in each experiment was determined to give an appropriate power of the test.<br>The sample sizes of different experiments, including statistics of pups weight and immunofluorescence microscopy, were chosen accord ing to the previous studies published by other laboratories.<br>For in vitro experiments, we took at least 6 replicated per experiment and then at least 3 independent experiments were performed. For animal experiment (survival analysis), at least 8 animals were used per group. For statistical immunological staining, at least 20 fields were used.
Data exclusions	No data were excluded from the experiments.
Replication	The experiments were performed to have three biological replicates independently with similar results. Statistical test were assessed to compare the means of each experiment with the 3 independent experiments. A nova test was used to compare multi-sample experiments and student test for comparing 2 conditions.
Randomization	Sample were allocated randomly to experimental groups, except when purpose of the experiment compare difference between controls and Fus-OE mice. In histological staining, more than 4 visual fields in each of the 5 directions of the upper, lower, left, right and middle were randomly selected for statistics.
Blinding	All experiments were conducted in a double blinded fashion in which the researchers were blinded to group allocation.<br>Blinded experiments were performed for genes expression analyses of human milk.

Reporting for specific materials, systems and methods

We require information from authors about some types of materials, experimental systems and methods used in many studies. Here, indicate whether each material, system or method listed is relevant to your study. If you are not sure if a list item applies to your research, read the appropriate section before selecting a response.

Materials & experimental systems

n/a	Involved in the study
	[x] Antibodies
	[x] Eukaryotic cell lines
[x]	Palaeontology and archaeology
	[x] Animals and other organisms
[x]	Clinical data
[x]	Dual use research of concern
[x]	Plants

Methods

n/a	Involved in the study
[x]	ChIP-seq
	[x] Flow cytometry
[x]	MRI-based neuroimaging

Antibodies

Antibodies used

IHC/IF
CD133 (1:100, ABclonal, #A0219)
EpCAM (1:100, Abcam, #ab71916)
K14 (1:50, Abcam, #ab118685)
K18 (1:50, Abcam, #ab133263)
milk (1:500, Nordic Immunology, #5941)
Ki67 (1:500, Abcam, #ab15580)
ELF5 (1:500, ABclonal, #A7181)
E-cadherin (1:100, Abcam, #ab40772)
Alexa Fluor 555 goat anti-rabbit IgG (1:500, Invitrogen, #A21428)
Alexa Fluor 488 goat anti-mouse IgG (1:500, Abcam, #ab150113)
anti-rabbit IgG (1:200, Abcam, #ab6721)
WB:
α-Tubulin (1:5 000, Sigma, #T5168)
GAPDH (1:2 000, Santa Cruz, #sc-25778)
Flag (1:2 000, CST, #14793)
Cre (1:1 000, Abcam, #ab190177)
GFP (1:1 000, Abcam, #ab290)
FUS (1:1 000, Abcam, #ab124923)
p57Kip2 (1:1 000, Abcam, #ab75974)
PCNA (1:1 000, Abcam, #ab29)
Cyclin B1 (1:1 000, Abcam, #ab72)
Cyclin D1 (1:1 000, CST, #2978S)
Cyclin E1 (1:1 000, CST, #4129S)
P21 (1:1 000, Abcam, #ab109199)
P27 (1:1 000, BD biosciences, #610241)
anti-mouse IgG (1: 5 000, Abcam, #ab6728)
anti-mouse IgG (1:5 000, Abcam, #ab6721)

Validation

Each antibody has been validated by the companies and by the result of the paper. CD133 (1:100, ABclonal, #A0219) was used for IF, and suitable for mice and sugar gliders, which had been validated at Feijen J., 2014 and our study. EpCAM (1:100, Abcam, #ab71916) was used for IF, and suitable for mice and sugar gliders, which had been validated at Lei Z et al, 2022 and our study. K14 (1:50, Abcam, #ab118685) and K18 (1:50, Abcam, #ab133263) were used for IF, and suitable for mice, and validated by Zhao et al., 2020. Ki67 (Abcam, ab15580) were used for IF and IHC, and suitable for mice, and validated by Zhao et al., 2020. milk (1:500, Nordic Immunology, #5941) were used for IF and IHC, and suitable for mice and sugar gliders, and validated by Zhao et al., 2020 and our study. ELF5 (1:500, ABclonal, #A7181) was used for IHC and IF, and suitable for mice, which had been validated by the companies and our study. E-cadherin (1:100, Abcam, #ab40772) was used for IF, and suitable for mice, which had been validated by the companies and our study. α- tubulin(Sigma, T5168) and Gapdh (1:2 000, Santa Cruz, #sc-25778) were used for WB, and suitable for mice and sugar gliders, which had been validated by the companies and our study. Flag (1:2 000, CST, #14793) was used for WB, and suitable for mice, which had been validated at Ma M et al, 2024 and our study. Cre (1:1 000, Abcam, #ab190177) was used for WB, and suitable for mice, which had been validated at Bok I et al, 2021 and our study. GFP (1:1 000, Abcam, #ab290) was used for WB, and suitable for mice, which had been validated by the companies and our study. FUS (Abcam, #ab124923) was used for IF IHC and WB, and suitable for mice and sugar gliders, which had been validated at Zhang Y et al, 2022 and our study. p57Kip2 (1:1 000, Abcam, #ab75974) was used for WB, and suitable for mice, which had been validated by the companies and our study. PCNA (1:1 000, Abcam, #ab29) was used for WB, and suitable for mice, which had been validated at Brossard C et al, 2023 and our study. Cyclin B1 (1:1 000, Abcam, #ab72) was used for WB, and suitable for mice, which had been validated by the companies and our study. Cyclin D1 (1:1 000, CST, #2978S) was used for WB, and suitable for mice, which had been validated at Chan CH et al, 2023 and our study. Cyclin E1 (1:1 000, CST, #4129S) was used for WB, and suitable for mice, which had been validated at de la Peña Avalos B et al, 2023 and our study. P21 (1:1 000, Abcam, #ab109199) was used for WB, and suitable for mice, which had been validated at Adili A et al, 2022 and our study. P27 (1:1 000, BD biosciences, #610241) was used for WB, and suitable for mice, which had been validated by the companies and our study.

Eukaryotic cell lines

Policy information about cell lines and Sex and Gender in Research

Cell line source(s)	HC11 cells obtained from the Bernd Groner Lab, Ludwig Institute for Cancer Research. HEK293T and MCF10A were purchased from ATCC.
Authentication	None of the cell line used have been authenticated.
Mycoplasma contamination	The HC11, HEK293T, and MCF-10A cell lines were tested negative for mycoplasma contamiation.
Commonly misidentified lines (See ICLAC register)	No misidentified cell line was used in this study.

Animals and other research organisms

Policy information about studies involving animals; ARRIVE guidelines recommended for reporting animal research, and Sex and Gender in Research

Laboratory animals	C57/B6 mice at 8 weeks were purchased from the Experimental Animal Center of the Kunming Institute of Zoology (China). Fus-OE mice at 4 weeks were purchased from the Biocytogen (China). The WAP-Cre and K14-CreERT mice at 8 weeks were obtained from the Jackson Laboratory (USA). The mice were raised in a specific pathogen free (SPF) environment with an ambient temperature of 18-22 °C, a humidity of 50%-60%, and a 12 h light-dark cycle. The sugar gliders at 8 mouths were obtained from the pet trade and thereafter were maintained in breeding colonies at the Kunming Institute of Zoology, Chinese Academy of Sciences.
Wild animals	Horseshoe bats at 10 mouths were captured in Yunnan, China, between 2022 and 2023. The bats were brought back to the laboratory, anesthetized with isoflurane and killed by neck amputation. Subcutaneous breast tissue was then taken. The body was soaked in formalin and then managed and disposed of by the animal center.
Reporting on sex	The study did not involve reporting on sex.
Field-collected samples	The study did not involve sample collected from the field.
Ethics oversight	All experimental procedures and animal (Fus-OE, WAP-Cre, and sugar gliders) care and handling were performed per the protocols approved by the Ethics Committee of the Kunming Institute of Zoology, Chinese Academy of Sciences (IACUC-PA-2023-03-049).

Note that full information on the approval of the study protocol must also be provided in the manuscript.

Plants

Seed stocks	N/A
Novel plant genotypes	N/A
Authentication	N/A

Flow Cytometry

Plots

Confirm that:

- [x] The axis labels state the marker and fluorochrome used (e.g. CD4-FITC).
- [x] The axis scales are clearly visible. Include numbers along axes only for bottom left plot of group (a 'group' is an analysis of identical markers).
- [x] All plots are contour plots with outliers or pseudocolor plots.
- [x] A numerical value for number of cells or percentage (with statistics) is provided.

Methodology

Sample preparation	HC11 and primary cells were cultured in complete medium for 24 h at 37℃ after growth in blank medium for 12 h at 37℃. The cells were immobilized with 75% ethanol overnight at 4°C, followed by incubation with propidium iodide (PI) mixture (0.1 mg/ml PI, 1 mg/ml RNase A, and 0.6% NP40) at 37℃ for 30 min away from light.

Instrument	BD LSRFortessa
Software	Flow jo
Cell population abundance	3 × 10^5 cells.
Gating strategy	Cells within a gate are single cell in the main cell population that has been stripped of fragments and adherent cells.

☒ Tick this box to confirm that a figure exemplifying the gating strategy is provided in the Supplementary Information.
